# Understanding Water Storage Practices of Urban Residents of an Endemic Dengue Area in Colombia: Perceptions, Rationale and Socio-Demographic Characteristics

**DOI:** 10.1371/journal.pone.0129054

**Published:** 2015-06-10

**Authors:** Tatiana García-Betancourt, Diana Rocío Higuera-Mendieta, Catalina González-Uribe, Sebastian Cortés, Juliana Quintero

**Affiliations:** Centro de Estudios e Investigación en Salud (CEIS), Fundación Santa Fe de Bogotá, Bogotá, Colombia; Cary Institute of Ecosystem Studies, UNITED STATES

## Abstract

**Introduction:**

The main preventive measure against dengue virus transmission is often based on actions to control *Ae*. *Aegypti* reproduction by targeting water containers of clean and stagnant water. Household water storage has received special attention in prevention strategies but the evidence about the rationale of this human practice is limited. The objective was to identify and describe water storage practices among residents of an urban area in Colombia (Girardot) and its association with reported perceptions, rationales and socio-demographic characteristics with a mixed methods approach.

**Methods:**

Knowledge, attitudes and practices and entomological surveys from 1,721 households and 26 semi-structured interviews were conducted among residents of Girardot and technicians of the local vector borne disease program. A multivariate analysis was performed to identify associations between a water storage practice and socio-demographic characteristics, and knowledge, attitudes and practices about dengue and immature forms of the vector, which were then triangulated with qualitative information.

**Results:**

Water storage is a cultural practice in Girardot. There are two main reasons for storage: The scarcity concern based on a long history of shortages of water in the region and the perception of high prices in water rates, contrary to what was reported by the local water company. The practice of water storage was associated with being a housewife (Inverse OR: 2.6, 95% CI 1.5 -4.3). The use of stored water depends on the type of container used, while water stored in *alberca* (Intra household cement basins) is mainly used for domestic cleaning chores, water in plastic containers is used for cooking.

**Conclusions:**

It is essential to understand social practices that can increase or reduce the number of breeding sites of *Ae*. *Aegypti*. Identification of individuals who store water and the rationale of such storage allow a better understanding of the social dynamics that lead to water accumulation.

## Introduction

### Dengue in the world and Colombia

Dengue is the most important mosquito borne viral disease and a major international public health concern. Over 2.5 billon people are at risk from dengue [1] and is endemic in more than 100 countries of Africa, the Americas, the Eastern Mediterranean, South-East Asia and the Western Pacific [2]. Its incidence has grown around the world in the last decade [3,4]. Additionally it was estimated that dengue produced 670 DALYS (Disability adjusted life years) worldwide in 2004 [5], the median cost of a reported dengue case was 1,031 dollars in Asia and the Americas [6]. Estimates suggest the average cost of dengue illness per year to be 2.1 billion dollars in the Americas [7].

Colombia is the eighth most highly endemic country worldwide for dengue, based on the average number of cases between 2004 and 2010 [8]. Twelve epidemics have occurred in the country between 1971 and 2010, accumulating more than one million dengue cases between 1990 and 2010 [9]. A major national epidemic was registered in 2010, with 157,203 cases; 147,426 dengue cases and 9,777 severe dengue, with lethality of 2.26% (230 deaths). During 2013 an important epidemiological outbreak was registered in the country. A total of 127,219 dengue cases were reported with an incidence rate of 476.2; 3,377 severe dengue cases and 161 deaths through the year [3]. *Ae*. *aegypti*, the main dengue vector in Colombia, inhabits approximately 95% of the national territory with high infestation rates in multiple cities located below 1.800 meters above sea level [9]. Out of 1,121, 811 municipalities are situated below this altitude, of which 758 (93%) have reported dengue cases between 1999 and 2010 [9]. The estimated population at risk is round 24 million [9].

Vector control is widely used as a preventive measure against dengue transmission [10,11]. Given that targeting water containers where *Ae*. *aegypti* breeds [12,13] effectively reduces the adult vector density, and that most of the breeding sites are usually derived from human activities [13,14], water storage has received special attention in prevention strategies [12,15]. Specifically in Colombia, recent studies have identified the most productive breeding sites (*albercas* and tanks) [16] and as a result national policies have enhanced their recommendations in lidding and washing these containers [17]. Nonetheless, research addressing water accumulation and dengue is limited.

According to the evidence, most studies treat water storage as an independent variable associated to immature forms of the vector [18–20], included as a score of practices in order to measure its relationship with knowledge [21,22], or reported as part of a descriptive study about knowledge, attitudes and practices regarding dengue [23–27]. Other studies have focused on water storage as a consequence of irregular water supply and its relationship to vector productivity [15, 28] or on different aspects of storage such as use, quality of water, emptying patterns, lidding [29–31] and costs [29]. There is a lack of studies about the perceptions, rationales and socio-demographic characteristics among the people who practice water storage. The aim of this study was to identify and describe water storage among urban residents of Girardot and its association with reported perceptions, rationale and socio-demographic characteristics from a mixed methods approach. Water storage is defined as the voluntary practice of saving water regardless of the type of container, duration and source of water.

## Materials and Methods

### Study area and population

Girardot is located 120 Km from Bogotá (Capital District of Colombia) with a population of 103,839 inhabitants [32]. In the last decade, the city has experienced rapid urbanization growth given its touristic status. In a regular weekend the floating population can reach half of its official census and it duplicates during holiday seasons [33]. Girardot has ideal climatic characteristics for dengue transmission with a mean temperature of 33.3°C with 66.38% humidity and two rainy seasons (March–May and October–November) [34]. The city is one of 19 municipalities that reported 50% of all dengue cases in the country during the last decade [9]. In 2013, the number of confirmed cases in Girardot until 19^th^ of October surpassed those recorded during the 2010 epidemic (822 vs 1,745) [35].

### Study type and data collection

The study was carried out in the context of a Cluster Randomized Trial to assess the effectiveness, feasibility and cost of a community based intervention for the reduction of vector density [16]. There were a total of 20 clusters; each of 100 households. For data collection we used a mixed methods approach. We used quantitative base-line data collected previous to the implementation of the intervention. A qualitative study was designed to gather information about water storage during the implementation of the intervention. Questionnaires were developed, adjusted and applied by an anthropologist and a general physician.

#### Quantitative data

Household KAP (knowledge attitudes and practice survey) and entomologic baseline surveys were collected in 1,825 households of Girardot during four months (Feb-Jun 2013) corresponding to dry and wet seasons. Out of this sample, 104 surveys were excluded from the statistical analysis due to: 1) information errors of 0.2% (4/1,825) regarding respondent’s identification and data error transfer of 5.5% (100/1,825) of surveys collected through the mobile application. The analytical sample was determined as 1,721 (94.3%) households with complete KAP and entomological available information. The household survey consisted of 84 self-reported questions divided in six sections (S1 Questionnaire): 1. Socio demographic characteristics of the respondent (age, sex, education, migration, household income, household size (number of persons), 2. Household characteristics (construction materials and access to public services) 3. Gender decision-making regarding health care of family members, household chores, maintenance and purchases 4. Family member’s information (age, sex, occupation and medical history of dengue) 5. Disease symptoms and signs, treatment, transmission and prevention and knowledge of vector characteristics and 6. Attitudes and practices regarding control and prevention of dengue and its vector. The practices domain contained a section regarding water storage that included the following variables: water sources, uses and frequency of water container emptying. The entomologic survey was registered through observation by trained personal. All potential water containers in the households were inspected using a previously validated form to collect information, for this study we only used the following variables of this form (type and size of container, amount of water and use) (S2 Questionnaire. Entomologic survey).

#### Qualitative data

We conducted 26 semi-structured interviews with twenty residents and six technicians of the vector borne control disease program (S1 Text), selected through snowball referral [36] (Table 1).

**Table 1:** Occupation and age of the interviewees.

	Gender	Total		Age			Reported Occupation		
			Average	Maximum	Minimum	Employee	Independent	Housewife	Pensioner
**Community**	Men	6	60	78	47	2	2	0	2
	Woman	14	55.6	77	40	2	4	7	1
**Technicians**	Men	4	53	65	37	4			
	Woman	2	38.5	39	38	2			

The number of interviews was established inductively based on theoretical saturation, when no new information was obtained [36]. Interviews were recorded and names were codified for the analysis. Community interviews enquired about household composition, characteristics of the neighborhood, water services (quality and service perception), water bill price, water service interruption, water storage (definition, per type of water, per type of container, reasons for water storage), reported perceptions for water storage rationale by neighbors and the relationship between water accumulation and dengue. Interviews with technicians enquired about the same categories as in the community questionnaire, but the questions were based on the perception and actions of the residents in the neighborhoods under surveillance by each technician. The instrument and sample assessment were discussed with the research team and confirmed through field observation.

#### Data analysis

A total of 1,721 (94.3%) households were selected as the analytical sample. In order to assess concordance between self-reported versus observed storage of water, a new variable was generated to identify households with water containers reported both in the KAP survey and confirmed from the entomological assessment. Therefore, only households with both KAP and entomological survey were included in the statistical analysis. The outcome of interest was water storage. Respondents were asked whether they stored water in their household (yes/no). Given the lower prevalence of non-water storage (4.8%) compared to water storage (95.2%), the variable was recoded for the purpose of the statistical analysis (no/yes) and inverse odds ratios (water storage yes/no) are reported for ease of interpretation. The analytical strategy consisted of two stages. First, a bivariate analyses was conducted in order to identify possible associations between water storage (no/yes) and socio demographic characteristics, knowledge, attitudes and practices regarding dengue and *Ae*. *aegypti*. Chi-squared and Fisher exact tests were performed following the distribution of the selected variables. Second, significant unadjusted associations at the 90% level were included in the regression analysis (Tables 2 and 3); no variable of relevance to the researchers with no statistical significance was excluded from the analysis. Three different logistic regression models were performed for water storage (no/yes) (Table 4): the first included only sociodemographic variables, the second model included KAP variables only and the third model was a fully adjusted model with both socio demographic and KAP variables. The mean variance inflation factor was calculated for each model to assess multicollinearity between socio demographic and knowledge variables. Separate models were estimated to inspect for differences in the coefficients across models in order to account for confounding. All statistical analyses were conducted using Stata version 12 (StataCorp LP, College station, TX, USA).

**Table 2:** Sociodemographic characteristics according to water storage, Girardot (n = 1,721).

Water Storage	*Yes*	*No*	*p* value
Age (average)	48	43	0.01 (a)
Years of education (average)	11	13	<0.01 (a)
Frequency	(n = 1,639)	(n = 82)	
Sex:			0.48 (b)
Female	1,179 (68.5%)	56 (3.3%)	
Male	460 (26.7%)	26 (1.5%)	
Ocupation:			<0.001 (c)
Worker	669 (38.9%)	56 (3.3%)	
Unemployed	53 (3.1%)	2 (0.1%)	
Student	52 (3%)	2 (0.1%)	
House-keeping	726 (42.2%)	21 (1.2%)	
Pensioned	109 (6.3%)	0 (0%)	
Other	30 (1.7%)	1 (0.1%)	
Income (d)			0.03 (b)
Less than 1 MW	744 (43.2%)	28 (1.6%)	
Between 1 and 2 MW	764 (44.4%)	43 (2.5%)	
More than 2 MW	86 (5%)	9 (0.5%)	
Number of habitants per household			0.2 (b)
1	210 (12.2%)	18 (1%)	
2	319 (18.5%)	17 (1%)	
3	390 (22.7%)	14 (0.8%)	
4	351 (20.4%)	15 (0.9%)	
5	196 (11.4%)	8 (0.5%)	
>6	173 (10.1%)	10 (0.6%)	

^(a)^ Wilcoxon rank-sum test.

^(b)^ Chi square test.

^(c)^ Fisher test.

^(d)^ 47 survey respondent did not answer this question.

MW: Minimum wage.

**Table 3:** Reported KAP variables regarding dengue and *Ae*. *aegypti* vector according to water storage, Girardot 2013 (n = 1,721).

Water Storage	Yes	No	*p* value
Frequency	(n = 1,639)	(n = 82)	
		Knowledge	
Have heard about dengue			0.07 (b)
Yes	1,609 (93.5%)	78 (4.5%)	
No	30 (1.7%)	4 (0.2%)	
Dengue transmission by mosquito bite			0.46 (a)
Yes	1,533 (89.1%)	75 (4.4%)	
No	106 (6.2%)	7 (0.4%)	
Vector oviposition in clean stagnant water			0.01 (a)
Yes	736 (42.8%)	25 (1.5%)	
No	903 (52.5%)	57 (3.3%)	
Vector color identification as black with white stripes			0.48 (a)
Yes	581 (33.8%)	26 (1.5%)	
No	1,058 (61.5%)	56 (3.3%)	
Actions in order to prevent dengue directed to immature forms of the vector			0.24 (a)
Yes	1,580 (91.8%	77 (4.5%)	
No	59 (3.4%)	5 (0.3%)	
Actions in order to prevent dengue directed to adults forms of the vector			0.02 (a)
Yes	276 (16%)	22 (1.3%)	
No	1,362 (79.1%)	60 (3.5%)	
Signs of disease (1)			
Fever	1,514 (88%)	73 (4.2%)	0.26 (a)
Headache	914 (53.1%)	39 (2.3%)	0.14 (a)
Bone pain	752 (43.7%)	32 (1.9%)	0.22 (a)
Muscle pain	349 (20.3%)	15 (0.9%)	0.51 (a)
Stomachache	288 (16.7%)	15 (0.9%)	0.86 (a)
Eye pain	271 (15.7%)	7 (0.4%)	0.05 (a)
Nausea	842 (48.9%)	41 (4.2%)	0.8 (a)
Diarrea	669 (38.9%)	27 (1.6%)	0.15 (a)
Diaphoresis	81 (4.7%)	2 (0.1%)	0.30 (a)
Petequiae	126 (7.3%)	5 (0.3%)	0.6 (a)
Bleeding gums	50 (2.9%)	2 (0.1%)	0.75 (a)
Bleeding nose	51 (3%)	0 (0%)	0.1 (a)
Weakness	231 (13.4%)	14 (0.8%)	0.45 (a)
Do not know	66 (3.8%)	3 (0.2%)	0.87 (a)
	Attitudes		
Dengue is a community problem			0.29 (b)
Yes	1,554 (90.3%)	78 (4.5%)	
No	80 (4.6%)	3 (0.2%)	
Do not know	5 (0.3%)	1 (0.1%)	
Dengue is a community problem			0.75 (b)
Yes	1,585 (92.1%)	79 (4.6%)	
No	54 (3.1%)	3 (0.2%)	
Opinion about dengue seriousness			0.91 (b)
Serious	1,483 (86.2%)	76 (4.4%)	
Moderate	26 (1.5%)	0 (0%)	
Minor	13 (0.8%)	0 (0%)	
All options	98 (5.7%)	5 (0.3%)	
Do not know	19 (1.1%)	1 (0.1%)	
	Practices		
Practices in order to prevent dengue directed to immature forms of the vector			0.19 (a)
Yes	546 (31.7%)	33 (1.9%)	
No	1093 (63.5%)	49 (2.8%)	
Practices in order to prevent dengue directed to adults forms of the vector			0.33 (a)
Yes	1,096 (63.7%)	59 (3.4%)	
No	543 (31.6%)	23 (1.3%)	
Practices in order to prevent dengue directed to adults or immature forms of the vector			0.15 (a)
Yes	404 (23.5%)	26 (1.5%)	
No	1,235 (71.8%)	56 (3.3%)	

^(a)^ Chi square test.

^(b)^ Fisher test.

**Table 4:** The effect of sociodemographic and KAP characteristics on the absence of water storage practices (a).

	Sociodemographic variables (OR)	(95% CI)	Knowledge variables (OR)	(95% CI)	Overall specification (OR)	(95% CI)
**Socio demographic variables**						
Age	1.00	(0.92–1.10)			1.02	(0.93–1.12)
Years of education	1.03	(0.84–1.25)			1.03	(0.84–1.27)
*Surveyed occupation*						
Worker	1				1	
Unemployed	0.49	(0.11–2.09)			0.48	(0.11–2.07)
Student	0.41	(0.09–1.88)			0.42	(0.09–1.96)
House-keeping	0.39***	(0.23–0.67)			0.39***	(0.22–0.67)
*Household Income*						
Less than 1 MW	1				1	
Between 1 and 2 MW	1.42	(0.86–2.34)			1.23	(0.73–2.09)
More than 2 MW	2.37	(1.00–5.64)			1.90	(0.78–4.63)
**Knowledge variables**						
Have heard about dengue: no			1		1	
Have heard about dengue: yes			0.35	(0.12–1.05)	0.22**	(0.07–0.71)
Vector oviposition in clean stagnant water: no			1		1	
Vector oviposition in clean stagnant water: yes			0.59**	(0.36–0.97)	0.6	(0.36–1.00)
Actions in order to prevent dengue directed to adults forms of the vector: no			1		1	
Actions in order to prevent dengue directed to adults forms of the vector: yes			1.71**	(1.02–2.87)	1.6	(0.93–2.75)
Constant	0.05**	(0.00–0.53)	0.15***	(0.05–0.42)	0.16	(0.01–2.03)
Observations	1,541 (a)		1,721		1,541 (a)	

*** p<0.01,

** p<0.05.

^(a)^ Odds ratios were estimated using a logistic regression, where the dependent variable is water storage practice (Absence of water storage = 1 and presence of water storage = 0).

^(b)^ The differences in N values are due to missing values in the income report (47) and to lack of variance in some categories of the surveyed occupation (Pensioned 109 and Other 24).

For qualitative data, information obtained from the interviews was organized according to the analytical categories including perception about water service, water bill price, water service interruptions, frequency and duration, actions during water interruptions and water storage definition and practices (S1 Table). Community perceptions on water service fees were compared to official information provided by the local water supply company.

#### Triangulation

In this study we used triangulation methods in order to increase confirmability and credibility of the results. Triangulation was defined as the process that combines different strategies to collect data and analyze it, to guarantee a better understating of one phenomenon [37,38].

**Fig 1:**
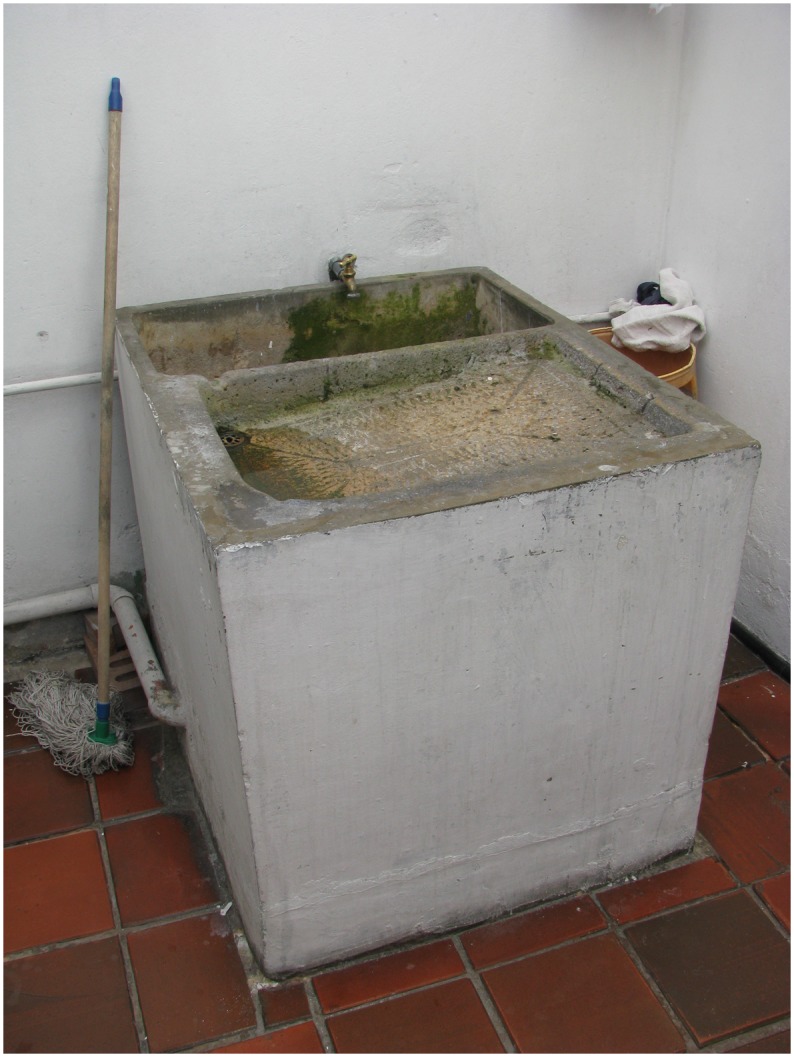
Alberca: Cement basins typically located in the laundry zone of the household with a capacity between 20 and 1000 liters.

In this project quantitative and qualitative data were gathered using the same analytical categories in order to have comparable and complementary information. The research team used all data collected to answer the following questions: Who stores water? What is this water used for? And why do people store water? In the process of triangulation, the research team decided to include a new category of analysis, which corresponds to the estimation of the costs of emptying water containers. Emptying and washing *albercas* (Fig 1), a key main breeding site, is one of the recommendations for dengue vector control in Colombia [17] therefore the economic cost of following this measure was estimated. Costs were calculated using the physical dimensions of *albercas* (cement basins typically located in the laundry zone of the household (Fig 1). Identified by entomological surveys and the basic fee per cubic meter provided by the local water supply company [39]. An average volume of *albercas* was calculated and then multiplied by the fee per cubic meter. Given that fees are differential according to socioeconomic strata (from 1 to 6 lower to higher), a differential cost was calculated for socioeconomic strata 2 and 3 (87.6% of the sample). For cost estimation we assumed that *albercas* were full and emptying them implies to remove all the water.

#### Ethical aspects

This study received clearance from the Ethical Review Committee of Fundación Santa Fe de Bogotá and the World Health Organization (WHO) under protocol ID: A90296. All surveys required written informant consent and written consent of a witness to assure that all information was provided under voluntary participation. Written consent was recorded on the mobile device used for survey collection. The Ethics Committee and the local Institutional Review Boards approved the consent procedure and its form.

## Results

1,721 households were successfully surveyed (S1 Dataset), the mean age of respondents was 48 years old 71.7% were women, 47% had achieved secondary education followed by elementary (28.1%). The most common activities reported during the last week were house-keeping (43.4%) and working (42.1%), 99.2% of the population is classified with low socioeconomic strata and 94.3% reported less than USD $664 as monthly income, 46.1% of residents reported an income of less than a minimum wage (US$ 332) (Table 2). Entomological surveys identified 4,268 (73.4%) containers with water out of 5,810 identified containers.

### Who stores water?

Based on the household survey, 82% (n = 1,411) of respondents reported water storage in their households. When compared with the entomological inspections, 14.1% (n = 244) individuals who did not report water storage were found to have containers with water and 1% (n = 16) of respondents who reported this practice, had no containers in their households at the time of the inspection, 2.9% (n = 66) neither reported water storage nor containers were found, 95.2% of the households had containers with stored water. Regarding socioeconomic variables, bivariate analysis showed associations between water storage (no/yes) and age, education, occupation, and household income. No significant differences were reported between water storage (no/yes) and sex or number of habitants per household (Table 2). However, we observed a negative trend between education level and water storage (p-value = 0.01), whereby individuals with higher levels of education had less chance to store water. Water storage was associated with knowledge about oviposition and actions against mosquitoes (e.g. fumigation, bed net and insects repellent usage), and having heard about dengue. Water storage was not associated with knowledge about transmission of the disease, immature preventive actions, diseases symptoms and attitudes and practices (Table 3).

We performed three logistic regressions. The first model included as independent variables only sociodemographic characteristics, the estimation showed that people whose occupation is housekeeping (Inverse OR: 2.6, 95% CI: 1.5–4.3) have almost three times more chance to store water compared to workers (Table 4). Other sociodemographic characteristics such as age, years of education and household income were not associated to water storage. The second logistic model considered only knowledge variables, this specification showed that individuals who know about *Ae*. *Aegypti* oviposition (Inverse OR: 1.7, 95% CI: 1–2.8) have higher odds of practicing water accumulation and that people who know about actions to prevent dengue directed to adult forms of the vector (OR 1.7, 95% CI: 1.02–2.87) have higher odds to not store water (Table 4). The third specification includes both sociodemographic and knowledge variables, this model showed that people whose occupation is housekeeping (Inverse: 2.6, 95% CI: 1.5–4.5) still have higher odds to store water, individuals who reported having heard about dengue (Inverse OR: 4.5, 95% CI: 1.4–14.2) have almost four times more chance to accumulate water (Table 4). We found no large differences in the estimated ORs across the three models suggesting that there is no confounding within sociodemographic and knowledge characteristics include in the third model.

### What is the stored water for?

**Fig 2:**
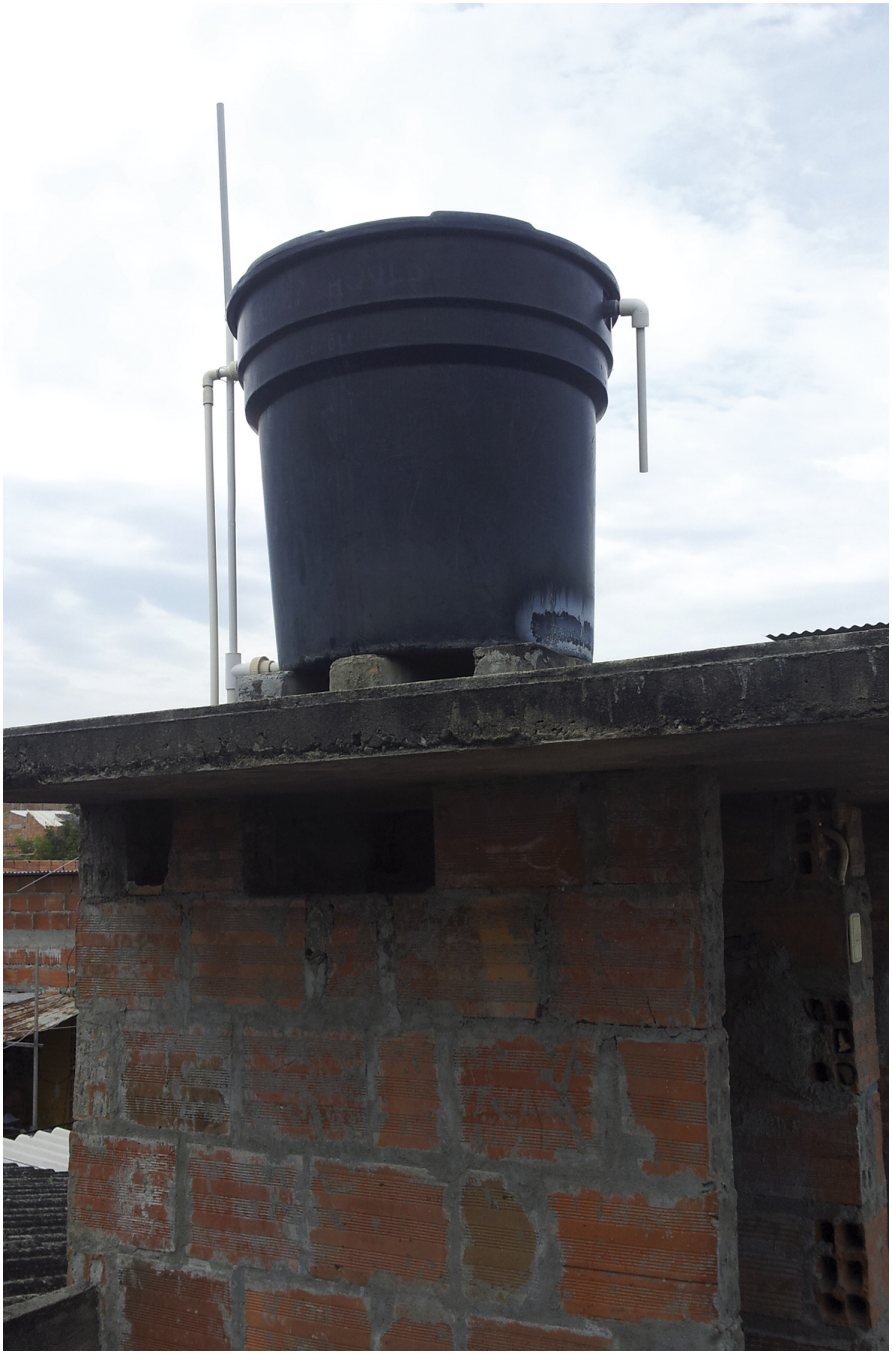
Tank: Cylindrical structure made of plastic or fibber cement with a capacity superior to 250 liters.

**Fig 3:**
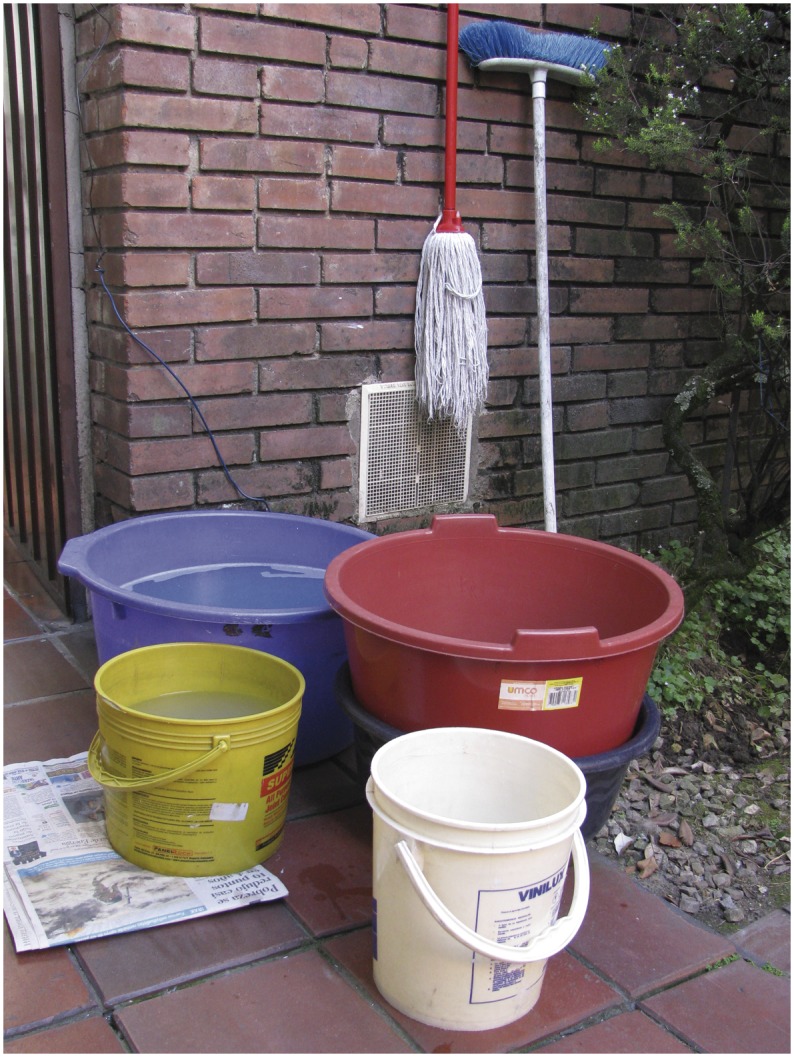
Plastic bins: Plastic containers with a capacity inferior or equal to 20 liters.

The most common containers for water storage reported by the community in the interviews (n = 20) were *albercas* (n = 14), tanks (n = 12) and plastic bins (n = 10) (Figs 1–3). The entomologic inspections showed that the most common containers were *albercas* (36.1%, n = 1,539), plastic bins (20.5% n = 877), bottles and recycled objects (14.7%, n = 629) and tanks (12.5% n = 532). The main use for storage water reported during the interviews was laundry and house cleaning, followed by “everything” and “for cloth and bath cleaning and cooking”. In the KAP survey, householders who use *alberca* reported as reasons for storage overall household cleaning 98% (n = 1,509), spare water 12.7% (n = 196) and human consumption 1% (n = 16). Technicians reported laundry as a unanimous use for this container.

Water storage in tanks (Fig 2) was reported as used for “everything” (n = 7 interviews). The tank is the principal deposit of water that provides water for bathrooms and *albercas*. Four residents reported that the tank supplied water for the bathroom, 3 for reserve, 2 exposed that the tank is empty and 1 used the water tank for house cleaning. Based on entomological inspections, water from tanks is used mainly for human consumption (54.3%, n = 289), as bathroom water supply (51.1%, n = 272) and reserve (31.4%, n = 167). Technicians reported during interviews that tanks are the main source of water supply for the bathroom.

Girardot has a particular bathroom architecture. Brick houses were constructed with a rectangular tank (>100 liters) above toilets and showers. The aqueduct pipe is connected to this tank through which all water is distributed to the bathroom. This system has been used to the present and sometimes replaces the use of plastic tanks above houses.

Eleven participants from the interviews used plastic bins (Fig 3). The reported use for water in this type of container included cooking (n = 4), water supply for the toilet (n = 2), cleaning (n = 1), cooking and cleaning (n = 1), washing (n = 1), watering plants (n = 1) and watering plants and cleaning (n = 1). Based on direct observation plastic bins are used as a reservoir in the kitchen in case of water shortage. During entomologic inspections, 73% (n = 641) of these containers were used for household cleaning, 15.3% (n = 134) for flushing the toilet, 10.6% (n = 93) for human consumption and 5.8% (n = 51) as spare water. Technicians reported cooking and watering plants as the main use for plastic bins during interviews.

### Why?

The reported rationale for water storage during community interviews (multiple choice answer) are water interruption services awareness (n = 13), reduction of the cost of the water bill (n = 6), and poor water pressure (n = 1); some participants could not separate the reason from the use of such storage (n = 3). In addition to what residents reported, all technicians when interviewed separately reported three principal reasons for water storage: water services interruptions, money saving and cultural tradition.

Awareness of water supply interruption of services leads to water accumulation as a preventive action; some participants argued the reason to store water is water service interruption during previous years.

*“In the past*, *we were often without water*. *Since two years ago*, *after improvements in pipe water distribution*, *we have regular water service”*

(Community member, 2014)

The principal reason for water storage was water cut services awareness, 14 participants stated this interruption and 6 exposed non-water service disturbances. Water services interruptions were reported with the following frequency; weekly (n = 3), monthly (n = 3), quarterly (n = 2), yearly (n = 1) and biyearly (n = 3). Two interviewees did not report a specific amount of time, one declared that water service interruptions were “a lot” and the other expressed “almost never”. Those who reported interruptions, informed that the water service was interrupted for 1 or 2 hours (n = 1) “less than a day” (n = 7), “for the day” (n = 5), and two days (n = 1).

The reported reasons for those interruptions include pipe maintenance or tank cleaning.

“*Water interruptions are notified by radio*, *the principal reason is maintenance*. *The aqueduct company is very careful*, *they program the interruptions and notify which districts will not have water and announce it on the radio (…) they alert five days earlier or so*. *Those interruptions last maximum 12 hours*, *never days*, *maximum 15 hours”* (Community member, 2014)

Another reason for water storage is cost saving due to high water costs (the average water bill reported was US$34.72) and also related to the perception that every single time they turn on the faucet, the meter starts to count. Locals perceived that the cost increases depending on the number of times they turn on the faucet instead of the amount of water used (cubic meters).

"If you are washing a dish and you open the faucet, that pressure makes the water reading to increase. Instead, if you have already collected water and you take it from a vessel, you save. Because even the air increases the water reading” (Community member, 2014)

### Water storage tradition

Acquisition of public services required several legal processes for illegal settlements like some neighborhoods of Girardot. This is the specific case of Pozo Azul, an unlicensed neighborhood that has been struggling to access public provision of piped water since its foundation in 2000. In the absence of proper and continuous water supply, the habitants firstly had to collect water from fire-engines that visited the settlement weekly, therefore many types of containers (pots, plastic bins and reusable bottles) were filled in order to have enough water for the week. Subsequently, a standpipe was installed and the community built a provisional network in order to distribute the water to all the houses. Nonetheless, given the poor quality of materials of such provisional network, shortages are common for repairing and maintenance leading to recurrent water storage. Gradually the number of households with intra-domiciliary water supply and a proper bill system has increased and consequently the number of households sharing the same pipe and bill has reduced to 40 out of 120 households nowadays.

“Previously when we suffered due to water shortage, we had to fill up even the last pot, for it to last at least two days” (Community member, 2014)

Furthermore, technicians (n = 6) argued that the main reasons for community water storage is cultural tradition (n = 4) followed by service interruptions (n = 2). Water management has changed through time in Girardot. Firstly, water was manually extracted from the river. When intra domiciliary water service was established (1960s) interruptions were frequent. As a consequence water storage became a necessity and developed into a tradition.

“Water storage is basically a custom, because our parents and grandparents did not have piped water (…) they began to store water out of need and to avoid travelling to the river daily, and these customs remain. With the development of aqueduct approximately 50 years ago people were already used to water storage and it proves to be a hard habit to break. People built albercas, because water supply was not reliable and sometimes water came dirty (…) with piped water it was even easier to collect it within the household increasing the number of containers for water storage”

(Technician, 2014)

### What does water storage mean?

25 out of 26 interviewees provided a definition for water storage without explaining its use. The community described it as an action, and used the words: “keep” “have” and “gather”. Among the places stated for this action, they identified plastic bins and tanks and some of them did not restrict their definition to a specific container. Additionally, four interviewees added covering as a condition for water storage and three included water supply interruptions.

“Having a vessel or a clean tank and having water and put a lid on”

(Community member, 2014)

All technicians defined water storage as an action and three of them also stated it was a cultural tradition. They used the words: “have”, “keep”, “fill” and “leave” and equally identified the containers. Only one specified its use and covering as other elements of the definition. One person was not able to give a definition of water without referring to its use.

### Estimation of the cost of emptying *albercas* weekly

Given that 87.6% (n = 1,601) of the sample resides in lower socioeconomic strata 2 and 3, the cost was calculated using the fees for these two levels. The average volume of *albercas* was 0.78 m^3^ for strata 2 and 0.67 m^3^ for strata 3 and the average cubic meter fee of water is US$ 0.55 and US $0.71 respectively (Exchange rate: $1,854 Colombian peso to 1 dollar. 9^th^ July 2014. Banco de la Republica) [39]. We estimated that if residents follow the recommendations of emptying a full tank and/or washing the *alberca* weekly, the monthly cost for implementing this specific dengue prevention practice would be of US$ 1.71 for strata 2 and US$1.90 for strata 3, 0.54% of the minimum national wage.

## Discussion

Water storage is a widely spread and culturally grounded practice in Girardot, 82% of the surveyed households reported this practice. Historically the main reason to store water is the apprehensiveness to interruptions in water supply rooted in a long history of water scarcity in the region that dates back to the 1960’s. Currently there is a perception of high price water fees that justifies water storage. The definition of water storage was different among community and technicians. Water storage practice was positively associated to being housekeeper, knowing about vector ovoposition and actions to prevent dengue directed to adult forms of the vector. Utilization of stored water depends on the type of container employed, while water in *albercas* is mainly used for household cleaning chores, water in plastic bins is used for cooking.

### Who store water?

Similar to what has been suggested in studies in Vietnam [40] and in Pakistan [27], in this study socio demographic characteristics are determinants of water storage, such as level of education, age and occupation, reported in the bivariate analysis. Housekeeping occupation was the only sociodemographic variable that remained significantly associated with water storage practice in the fully adjusted model. Associations between water storage practices and other sociodemographic and KAP variables were not found when controlling for KAP variables; this is due to the large reported prevalence of water storage. As it was found by Whiteford in Dominican Republic [41], and by Naing in Pakistan [21] water storage in Girardot plays an important role in household chores, such as laundry, cleaning and cooking which is why it was expected that individuals dedicated to such labors were more prone to report water storage. Additionally, as previously presented by other authors [18,20,24,26], having knowledge about mosquitoes and dengue does not lead to relatively less reported water storage behavior.

### Why: A culturally engrained behavior

Our study supports the hypothesis by Nalongsack [24] and Pacheco [26] that the availability of piped water does not entirely determine water storage. The tradition of water storage lies in four groups of determinants 1. Human perceptions 2. Sociocultural dynamics [30]; 3. Economic; and 4. Environmental factors [42] such as cultural traditions, high prices of water services bill, constant access to water [15], urbanization, tourism and climate. Therefore, we cannot separate the context from the practice. Water storage in Girardot stems from several kinds of factors such as a large history of water shortages; irregular water supply; the river as the only source of water and alternative methods to achieve water access in illegal settlements without provision of public services. All of these factors are part of the context that makes Girardot prone to water storage. Consequently, contextual factors have to be inquired further not only to understand storage practices but also to achieve control and prevention activities coherent with the resident’s behaviors and social context [43].

Water is essential for daily functions; therefore practices to ensure its availability and access have become indispensable in a context without regular water supply [2,15,43]. This study evidenced a large history of absence and interruptions of water service. Consequently, residents are accustomed to store water for cooking and cleaning congruent with Suarez’ findings in Girardot and Melgar [43]. Furthermore, individuals store despite having an uninterrupted water supply. Residents and technicians reported a regular service with few and previously notified interruptions. Nonetheless, when they were asked about the reason for this accumulation, they argued service disruption. Population have developed social imaginaries of water interruption based on historical lack of the service [44] thus a tradition of water storage has been rooted as found by Pacheco [26] and Nalongsack [24] in La Dorada, Colombia and Champasack in Laos respectively.

Residents of Girardot have a perception about high prices of public services, specifically water bill. Contrary to this, the Domiciliary Public Services Superintendence, the national authority for public service surveillance [45], reported that water service payment rates in Girardot were relatively lower than the national average except for strata 1 in which the fee is 11% higher than the national average (Strata 5 and 6 were excluded from the analysis). Interviewers reported a monthly average water bill cost that represents around 10% of the minimum wage representing a high economic burden for the household. Results suggest a contradiction between community perception about water price and low rates reported by the national and local authorities. Therefore, it is importance to understand the rationale of complex behaviors, such as water storage related to social and economic drivers.

### What does water storage mean? Not everyone on the same page

There is a difference between technicians and community’s definition of water storage. The community did not identify *albercas* as a container for water storage whereas technicians describe it as the key container. The community perceives *albercas* as a container where water is placed (not stored) to carry out household chores; this was evidenced by the divergence between self-reported water storage in the household survey and the finding of *albercas* with water in the entomological survey. Further research is needed given that the authors do not know if previews studies have assessed community and local authorities’ concepts of water storage before. This results are relevant given that previous studies showed that *albercas* are the main breeding-site therefore they are the main target of Dengue prevention and control strategies locally and nationally [46].

The use and rationale of water storage were challenging to disentangle. Individuals were not able to give a definition or offer a reason for water storage without explaining its use. This suggests the importance of understanding water storage as a complex activity that needs to be assessed from its use and rationale at the same time. Besides from this paper, Padmanabha, Tran and Subbaraman [29–31] have tried to consider it in such way, nonetheless it is required to be taken into account in the design of vector control strategies [2]. For example, households surveys (Knowledge Attitudes and Practices) could enquire about the meaning and relative importance of water storage in the local context.

### Understanding local water storage practices leads to integral preventive strategies against dengue

One of the main concerns of health authorities is the low prevalence of washing or emptying water containers. As reported by the community in other studies, cleaning tanks and *albercas* involves the use of large volumes of water and therefore decreasing washing frequency (more than 7 days) can save water costs [43]. In contrast, this study shows that the amount of water and money used in order to accomplish this recommendation is less than 0.54% of the current minimum wage. Thus, it is necessary to assess the cost of the control and prevention interventions that are going to be suggested or applied to a community in order to establish a dialogue with them based on their economic concerns as well.

Furthermore, community dengue interventions need to be based on the identification of social dynamics, population characteristics, motivations, behaviors, environmental interaction, social experience, historical [42,43] and political context [15,43]. Public policy has ignored some of these elements, concentrating its efforts in the measurement of entomological variables [9,30]. Understanding water storage and its links with such factors is an advance for the construction of concrete and coherent solutions [43] that can be developed on dengue public policies. Therefore studies assessing this problematic could enhance integral strategies for dengue prevention.

This study provides evidence of the importance of enquiring about the rationale for water storage in the local context. We suggest that further studies addressing behavioral aspects of dengue take into account water storage practices. Further research is needed regarding the incidence of public services rates and the burden in family income and water storage practices.

### Strengths and limitations

This study combined quantitative and qualitative methodologies for data gathering and analysis that allowed the comparison of several sources of information integrating different points of view. Mixed methods approach promotes fluent dialogue with the community and a better understanding of water accumulation, not only as a practice per se but also to the subject who performs the action. Among the limitations of the study, the lack of subjects who did not store water in the interviews did not allow us to assess the reasons for not storing water. Nonetheless despite of its low number, the individuals who did not report water storage were part of the quantitative analysis.

## Conclusions

Water storage is a common practice regardless of socioeconomic variables. The main reasons for this practice are the apprehensiveness about: interruptions in water supply that are almost non-existent nowadays and the high prices of water bill in the local context under study. Finally, before tackling water storage in household water containers it is necessary to identify community water storage definitions to establish a congruent communication between them and policy makers to accomplish the same results towards dengue vector control.

## Supporting Information

S1 Dataset (XLSX)

S1 Questionnaire Knowledge, attitudes and practice (KAP) survey. (XLSX)

S2 Questionnaire Entomologic survey. (XLSX)

S1 Table Qualitative categorical data. (XLSX)

S1 Text Interview question guideline. (DOCX)
