# Supplementary material for: Understanding Water Storage Practices of Urban Residents of an Endemic Dengue Area in Colombia: Perceptions, Rationale and Socio-Demographic Characteristics
Source: PLoS One. 2015 Jun 10;10(6):e0129054. doi: 10.1371/journal.pone.0129054 (PMC4465337; doi:10.1371/journal.pone.0129054)
Supplement: S1 Text — (DOCX) [file pone.0129054.s005.docx]

GUIA DE ENTREVISTA- COMUNIDAD

Nombre____________________________________________________________________________________________________________________

Barrio y Dirección_________________________________________________________________________________________________________

Conglomerado_____________________________________________________________________________________________________________

Ocupación__________________________________________________________________________________________________________________

Edad________________________________________________________________________________________________________________________

Entrevistador______________________________________________________________________________________________________________

Fecha_______________________________________________________________________________________________________________________

Numero de Entrevistas___________________________________________________________________________________________________

1. Cuénteme un poco de su hogar
2. ¿Quiénes viven aquí? ¿Edad? (Todas las personas que están en edad productiva trabajan, discapacitado, adulto mayor) No personas ____ Edades __________
3. ¿Todos los integrantes de su hogar nacieron aquí?
4. ¿A qué se dedica?
5. ¿Quién se encarga del cuidado de la casa?
6. ¿Hasta qué nivel de estudio ha alcanzado?
7. ¿Qué tan frecuente son los cortes de agua?
8. ¿Tiene servicio de acueducto y alcantarillado?
9. ¿Cómo le parece este servicio?
10. ¿Cómo le parece el precio de la factura del agua? ¿Por cuánto le llega?
11. ¿Cómo es la calidad del agua del acueducto?
12. ¿En este sector cortan el agua? ¿Por qué lo hacen? ¿Cada cuánto?
13. Cuando le cortan el agua ¿Usted qué hace?
14. Almacenamiento de agua
15. ¿Cómo define almacenamiento de agua?
16. ¿Usted almacena agua? ¿En dónde?
17. ¿Qué agua almacena?
18. Cuál es la razón de cada uno de los depósitos

| DEPOSITO | UBICACION | RAZON ¿PARA QUE? | FRECUENCIA VACIAMIENTO | ADICIONAL | TIPO DE AGUA | VENTAJAS Y DESVENTAJAS AGUA |
| --- | --- | --- | --- | --- | --- | --- |
|  |  |  |  |  |  |  |
|  |  |  |  |  |  |  |
|  |  |  |  |  |  |  |

1. ¿Porque usted almacena agua? (Tener en mente responder por qué y los elementos de cada razón)
2. Nos encontramos una casa donde tienen muchos (baldes, albercas, canecas, botellas) con agua ¿Por qué cree que la gente almacena esa agua?
3. ¿Si no hubiera cortes de agua usted almacenaría de agua?
4. Explorar otras categorías que salgan (IMAGINACION DEL ENTREVISTADOR)
5. Dengue
6. ¿Qué relación tiene el almacenamiento de agua y el dengue?
7. ¿Para usted que es el dengue?

GUIA DE ENTREVISTA-TECNICOS

Nombre____________________________________________________________________________________________________________________

Años en ETV_________________________________________________________________________________________________________

Conglomerado que tiene a cargo_________________________________________________________________________________________

Cargo__________________________________________________________________________________________________________________

Edad________________________________________________________________________________________________________________________

Entrevistador______________________________________________________________________________________________________________

Fecha_______________________________________________________________________________________________________________________

Numero de Entrevistas___________________________________________________________________________________________________

1. Cuénteme un de los conglomerados/sector de los que esta cargo
2. ¿Cuáles son las características de esos barrios? (estrato, tiempo de construcción, problemas mas frecuentes)
3. ¿Tiene servicio de acueducto y alcantarillado?
4. ¿Cómo le parece este servicio?
5. ¿Cómo es la calidad del agua del acueducto?
6. ¿Cuál es la opinión de la gente sobre la tarifa?
7. ¿Qué tan frecuente son los cortes de agua en este sector?
8. ¿En este sector cortan el agua? ¿Por qué lo hacen? ¿Cada cuánto?
9. Almacenamiento de agua
10. ¿Cómo define almacenamiento de agua?
11. ¿En el sector se almacena agua? ¿En dónde?
12. ¿Qué agua se almacena?
13. Cuál es la razón de cada uno de los depósitos

| DEPOSITO MAS FRECUENTES | RAZON ¿PARA QUE? MF | FRECUENCIA VACIAMIENTO MF | ADICIONAL | TIPO DE AGUA MF | VENTAJAS Y DESVENTAJAS AGUA MF |
| --- | --- | --- | --- | --- | --- |
|  |  |  |  |  |  |
|  |  |  |  |  |  |
|  |  |  |  |  |  |

1. ¿Porque la gente almacena agua? (Tener en mente responder por qué y los elementos de cada razón)
2. Nos encontramos una casa donde tienen muchos (baldes, albercas, canecas, botellas) con agua ¿Por qué cree que la gente almacena esa agua?
3. ¿Si no hubiera cortes de agua usted cree que la gente almacenaría agua?
4. Explorar otras categorías que salgan (IMAGINACION DEL ENTREVISTADOR)
5. Dengue
6. ¿Qué relación tiene el almacenamiento de agua y el dengue?
7. ¿Para usted que es el dengue?
